# Variations on the bilingual advantage? Links of Chinese and English proficiency to Chinese American children's self-regulation

**DOI:** 10.3389/fpsyg.2014.01069

**Published:** 2014-09-30

**Authors:** Stephen H. Chen, Qing Zhou, Yuuko Uchikoshi, Silvia A. Bunge

**Affiliations:** ^1^Department of Psychology, Wellesley CollegeWellesley, MA, USA; ^2^Department of Psychology, University of California, BerkeleyBerkeley, CA, USA; ^3^School of Education, University of California, DavisDavis, CA, USA

**Keywords:** bilingualism, self-regulation, immigrant children

## Abstract

The present study examined whether bilingualism-related advantages in self-regulation could be observed: (a) among Chinese American immigrant children with varying levels of Chinese and English proficiencies, and (b) across different domains of self-regulation in laboratory, home, and classroom contexts. A socioeconomically diverse sample of first- and second-generation Chinese American immigrant children between ages 7 and 10 (*n* = 223) was administered assessments of Chinese and English language proficiencies and a multi-method, multi-informant battery of self-regulation measures. Multiple regression analyses suggested that controlling for covariates (child age, gender, and SES), children's bilingualism-related advantages were limited to higher performance only on computerized tasks of cognitive flexibility, and only among children with higher degrees of fluency in both Chinese and English. By contrast, proficiencies in one language (either Chinese or English) were uniquely and positively associated with other domains of self-regulation, including parent and teacher-reported effortful control. These results suggest that the bilingual advantage for self-regulation may be observed as a continuous variable among immigrant children with varying levels of bilingual fluency; however, this advantage may not extend across all domains and contexts of self-regulation.

## Introduction

Though previous research suggests that bilingual experience may confer advantages for children's executive functioning (see Bialystok et al., 2009, for a review), recent findings indicate mixed support for the “bilingual advantage” (Antón et al., 2014; Gathercole et al., 2014; Pelham and Abrams, 2014). A more nuanced understanding of the bilingual advantage may be achieved by examining two questions that to-date remain largely unexplored. The first is whether bilingual advantages in executive functioning can be observed as a continuous variable. That is, among children who are bilingual or learning two languages, is executive functioning related to the degree of dual language proficiency? Second, previous research on the bilingual advantage has been limited largely to laboratory-based tasks of executive functioning. Can bilingualism-related advantages also be found in other domains of self-regulation (e.g., effortful control) or in other contexts (e.g., home and school)? We investigated these questions using a sample of Chinese American immigrant children. Specifically, we tested: (a) whether English and Chinese proficiency uniquely predict higher self-regulation among English-Chinese bilingual children, and (b) whether English and Chinese proficiency interact with each other in predicting children's self-regulation.

Executive function refers to higher-order, self-regulatory cognitive processes that aid in the monitoring and control of thought and action (Zelazo and Cunningham, 2007). Research to date on bilingualism-related advantages in children's executive function has operationalized “bilingualism” primarily as a categorical variable, and has compared bilingual children with monolingual peers on measures of executive function. One important limitation of the between-groups design is that classifying bilingual children into a single group masks the heterogeneity within the bilingual population. Children exposed to and learning two languages (i.e., dual language learners, or DLL) have considerable variability in their development of L1 and L2 (Hammer et al., 2011, 2014). For example, research with Cantonese-English bilinguals has shown large individual variation in language and early literacy measures for both children's home language (L1; Cantonese) and their school language (L2; English) (Uchikoshi and Marinova-Todd, 2012; Uchikoshi, 2013). This variation has led to differences in scores of more than one standard deviation, especially in their L1 Cantonese. This variation may be due to home language use, as well as supplemental instruction through bilingual programs or Saturday heritage language schools (Leung and Uchikoshi, 2012; Uchikoshi and Marinova-Todd, 2012; Uchikoshi, 2013).

Indeed, researchers have suggested that truly balanced bilinguals (i.e., individuals with equal competence in both languages) are rare, and that bilingual proficiency is more accurately represented on a continuum (Valdés, 2001). Some researchers have used a more nuanced approach to assess proficiencies in both languages, and quantify bilingualism as multiplicative terms of these proficiencies (Carlisle et al., 1999; San Francisco et al., 2006). Thus, by assessing and analyzing language proficiency as continuous variables, it is possible to examine the more complex relations between bilingualism and executive function. First, one can test whether proficiency in two languages uniquely relates to efficiencies in executive functioning. Because research has shown that proficiencies in two languages are correlated with each other in complex ways among bilingual children or DLLs (Hammer et al., 2014), it remains unknown whether proficiency in each language uniquely contributes to efficiency in executive function skills. Second, testing the interactive relation between two languages allows researchers to study whether children's executive function skills are related to their proficiency in L1 and L2. For example, do bilingual children who are proficient in both languages (i.e., balanced bilinguals) show higher executive function than those who are proficient in only one language (e.g., L1-dominant bilinguals, or L2-dominant bilinguals)?

Previous work on the bilingual advantage in executive function has primarily used lab-based cognitive measures, and there have been a number of findings that support bilingual children performing better on these tasks compared to monolingual children. Compared to monolingual children, balanced bilinguals performed significantly better on executive function tasks that require inhibiting distracting information (interference suppression), switching between tasks, working memory, or coordinating different executive control skills (Carlson and Meltzoff, 2008; Martin-Rhee and Bialystok, 2008; Bialystok, 2011; Yang et al., 2011). This advantage has been attributed to bilinguals' management of ongoing linguistic experience, which involves the constant recruitment of control processes to maintain focus on the language in current use while temporarily inhibiting access to the other language. The constant need to exercise executive function, in turn, is hypothesized to contribute to enhancement in these cognitive skills over time (Bialystok et al., 2009).

What remains unknown is whether the bilingual advantage on children's executive function skills can also be observed in other domains of children's self-regulation, namely, the regulation of emotion and behavior in natural contexts such as home and school. Recent theoretical and empirical work has suggested that executive function shares commonalities in its conceptualization, measurement, and neural structure with the temperament-based construct of effortful control. Effortful control refers to the voluntary inhibition of a prepotent, dominant response in order to activate a subdominant response (Rothbart and Bates, 2006; Blair and Razza, 2007; Zhou et al., 2012). Both executive function and effortful control can be conceptualized as self-regulatory processes associated with goal-directed behavior, and studies using both effortful control and executive function measures have suggested that these measures are modestly correlated and load on a common factor of self-regulation (Blair and Razza, 2007; Chen et al., 2014). However, while executive function has been conceptualized as involving primarily cognitive processes, and is typically measured using laboratory-based measures, effortful control is conceptualized as including more emotional regulatory processes, and is commonly assessed with parents' reports, teachers' reports, or tasks requiring behavioral persistence (Blair and Razza, 2007).

There are reasons to expect that DLLs may exhibit higher self-regulation beyond laboratory contexts. For example, it is possible that the attentional processes enhanced through bilingual experience may also be used to maintain focus in both home and classroom settings. Likewise, the ability to cognitively inhibit distracting information may also be taxed on behavioral tasks requiring persistence in the face of temptation.

It is also possible that the effects of bilingualism may extend beyond purely cognitive processes of executive function to more socioemotional processes involved in effortful control. Though we are unaware of research to-date specifically examining effects of bilingualism on effortful control, research with bicultural individuals suggests that switching regularly between cultural frames of reference allows one to adapt readily to changing social contexts and situations, and to develop more complex and nuanced representations of culture and the self (Benet-Martínez et al., 2002, 2006; Nguyen and Benet-Martínez, 2013). This higher self-complexity among bicultural individuals, in turn, has been theorized to buffer against negative stressors and emotional states (Linville, 1985; Yip, 2009). In support of this theory, a recent study by Yip (2009) indicated that among Chinese American undergraduates, simultaneous salience of both Chinese and American identities was associated with increased positive mood. Thus, as a form of biculturalism, bilingual experience may provide DLLs with increased adaptability and self-regulation in response to socioemotional stressors.

Alternatively, it is possible that higher self-regulation in these domains may be influenced by higher proficiency in one, rather than multiple, languages. Specifically, in the present study, children's higher Chinese proficiency may reflect greater orientation toward Chinese culture and its expectations of behavioral control and self-restraint, and may therefore be reflected in higher self-regulation across different domains (Chen et al., 1998; Jose et al., 2000; Luo et al., 2013). In support of this hypothesis, children from East Asian countries have been found to outperform European American children on tasks of executive function (Sabbagh et al., 2006; Oh and Lewis, 2008; Lan et al., 2011). Moreover, Asian-English bilinguals have been found to outperform Spanish-English and French-English bilinguals on tasks of attentional control (Yang and Lust, 2007).

Taken together, examination of the bilingual advantage in DLLs must consider both the interactive and unique effects of language proficiencies across different domains of self-regulation. First- and second-generation Chinese American immigrant children are an ideal sample in which to examine the relations between bilingualism and self-regulation. Chinese Americans are the largest subgroup of Asian Americans (23.5% of the Asian American population) and one of the fastest growing foreign-born populations in the United States (Pew Research Center., 2012). Moreover, Chinese American children have varying degrees of proficiency in English and Chinese (Leung and Uchikoshi, 2012; Uchikoshi and Marinova-Todd, 2012). For example, one study using cluster analysis found four distinct groups of young bilinguals: (a) children who had similar levels of English and Chinese vocabulary—i.e., balanced bilinguals; (b) higher English vocabulary and lower Chinese vocabulary (English-dominant); (c) high English vocabulary and high Cantonese vocabulary—so-called “ideal bilinguals”; and (d) higher Cantonese vocabulary and lower English vocabulary (Cantonese-dominant) (Leung and Uchikoshi, 2012). Moreover, previous research indicates that only 16.1% of second-generation immigrant youth are highly proficient in their family's heritage language, and 72.3% prefer speaking in English compared to their family's heritage language (Portes and Hao, 1998).

The present study tested the unique and interactive relations of Chinese American immigrant children's Chinese and English proficiencies to their self-regulation assessed by a multi-method battery. As some researchers have argued that SES and ethnicity are potential confounds in the associations between bilingualism and executive function (Morton and Harper, 2007), we examined the relations between bilingualism and self-regulation in a single-ethnicity sample of immigrant children, and controlled for family SES in the analyses. Based on the literature on the bilingual advantage in children's executive control (Bialystok, 2011), we hypothesized that proficiency in two languages would additively and interactively predict higher self-regulatory skills controlling for family SES and child demographics (age and gender). Given traditional emphasis of Chinese culture on children's behavioral control and self-restraint (Luo et al., 2013), we also expected that children's higher Chinese proficiency would be uniquely associated with their self-regulatory skills. Consistent with integrated models of self-regulation (Zhou et al., 2012; Chen et al., 2014), we also hypothesized that these associations would be observed across domains (executive function and effortful control) and contexts (laboratory, home, and school) of self-regulation.

## Methods

### Participants

Children were selected from a larger longitudinal study of Chinese American immigrant families in the Western United States. Children were either first-generation (born outside of the United States) or second-generation immigrants (born in the United States to at least one foreign-born parent). Data for the present study were collected at the second wave of the longitudinal assessment, when children (*n* = 239) were between 7 and 10 years of age.

To exclude monolingual Chinese or English speakers from the present sample, children were excluded from the present analyses if they: (a) scored more than one standard deviation below standard mean scores on a standardized test of English reading skills, or (b) were unable to identify at least one item on a test of Chinese receptive vocabulary (PPVT-R in Chinese; Lu and Liu, 1998). Children were administered the Basic Reading Skills Cluster of the Woodcock-Johnson Tests of Achievement, 3rd Edition (WJ-III), which consists of the Letter-Word Identification (naming and reading words from a list) and Word Attack subtests (reading non-words). Children who scored more than one standard deviation below the mean score on the WJ-III were excluded from analyses (*n* = 1). Children were also administered the Chinese version of the Peabody-Picture Vocabulary Test-Revised (Lu and Liu, 1998). Fourteen tests were invalid due to administration errors, and children who were unable to identify at least one item were excluded from analyses (*n* = 1).

The resulting sample included 223 Chinese American immigrant children, of whom 23.3% were first generation and 76.7% were second-generation immigrants. Approximately half (48.9%) of the children were girls. Children ranged from 7.49 to 10.96 years of age (*M* = 9.18, *SD* = 0.74). Almost all participating parents (80.3% mothers) were born outside of the United States: 74.0% of parents were born in mainland China, 8.5% were born in Hong Kong, 3.1% were born in Taiwan, and 12.1% were born in other parts of the world. Participating parents' years of formal education ranged from 2 to more than 20 years (Doctorate or other advanced degree). On average, participating parents had completed some post-secondary education (*M* = 13.22 years, *SD* = 2.87). Families' household per capita income was calculated by dividing the estimated total family income for the past year by the number of individuals living in the household (Datta and Meerman, 1980). Families' per capita income ranged from $1000 to over $33,750 (*M* = $11,841.49, *SD* = $8309.48). More than half the children in the present sample (57.3%) were eligible for free or reduced school lunch based on family income.

### Procedures

All participants were treated in accordance with ethical standards of APA. All procedures were approved by the University of California, Berkeley's Institutional Review Board and the Committee on the Protection of Human Subjects. Following informed consent procedures, the child and the participating parent participated in a 2.5-h laboratory assessment, which included structured interviews, questionnaires, cognitive and behavioral tasks, and tests of academic achievement. All questionnaires and tests were administered in the parent's or child's preferred language (English, Mandarin, or Cantonese) indicated at the beginning of the visit. All written materials, including consent and assent forms and questionnaires, were available in English, simplified Chinese, or traditional Chinese. The majority of parents (82.5%) completed the questionnaires in Chinese. All children in the present sample completed the assessment in English. At the end of the laboratory visit, parents were paid $50 and reimbursed for transportation, and children were given a small prize. At the end of data collection, a brief written feedback report was mailed to the parent, summarizing the child's performance on academic test and his/her overall emotional and behavioral adjustment, based on parent's and teacher's ratings on standardized instruments. After obtaining the parent's written permission, the child's main classroom teacher was contacted by the research staff and asked to complete a set of questionnaires on the child's behaviors at school. Teacher questionnaires were sent and returned via mail, and teachers were paid $20.00 for completing the questionnaires for each child. Teacher questionnaires were collected for 87.0% of children.

### Measures

We used a variety of laboratory tasks (observed behavioral measures, computerized tasks), parent questionnaires, and teacher questionnaires to assess four components of children's self-regulation: behavioral regulation, attention focusing, inhibitory control, and cognitive flexibility. Children's Chinese and English vocabularies were assessed through three laboratory assessments.

#### Puzzle box test of behavioral regulation

Children's behavioral regulation was assessed via their performance on a task in which they were required to assemble a puzzle without looking (Eisenberg et al., 2001, 2005). Children were presented with a wooden puzzle placed inside a large wooden box. The back of the box was covered with clear Plexiglas through which children's hand movements could be observed. The front of the box was covered by a black cloth, and the cloth had sleeves through which the children placed their arms. Although the cloth blocked the child's view of the puzzle, children were shown that they could easily “peek” during the task by lifting the cloth, but were instructed not to do so. Children were told that they had 5 min to complete the puzzle without looking, and were told that they would receive a prize for completing the puzzle. Children were left alone in the room for up to 5 min while completing the task and were videotaped by a visible video camera.

Two trained undergraduate students independently coded the videos for the number of seconds the child persisted on the puzzle without cheating or going off-task (inter-rater *r* = 0.97 in this sample). Children's behavioral persistence was calculated as the proportion of time persisting on the task (i.e., time persisting divided by the total time spent on the puzzle, see Eisenberg et al., 2001). In a longitudinal study of predominantly European American children, the behavioral persistence score on this task showed satisfactory rank order stability from middle to late childhood and loaded positively on the latent factor of effortful control together with parent and teacher report of attention focusing and inhibitory control (Eisenberg et al., 2005).

#### Computerized tests of response inhibition and cognitive flexibility

Response inhibition has been conceptualized as the ability to refrain from a prepotent or automatically-cued response, while interference suppression involves attending to a relevant cue while filtering out irrelevant and potentially conflicting cues (Bunge et al., 2002). Bilinguals have been shown to outperform monolinguals on tests of interference suppression, but no consistent advantages have been observed for tasks of response inhibition (Bialystok et al., 2006; Costa et al., 2008).

***Response inhibition task***. Children performed a computerized Go/No-Go task (Eriksen and Eriksen, 1974) designed for use in children. Children were presented with a series of images of cartoon characters from a popular animated children's film, and were instructed to press a button quickly in response to the appearance of a target stimulus, and to refrain from pressing the button in response to the appearance of a non-target stimulus. The stimuli were presented for 2 s each at consistent intervals. Designation of cartoons as either target or non-target stimuli were counterbalanced for each child. During an initial instructional phase consisting of 30 trials, the experimenter provided correctional feedback if the child made an error in responding. In the subsequent testing phase, no feedback was provided. Each testing phase consisted of 200 target/Go trials and 50 non-target/No-Go trials. Data from the testing phase were submitted for analysis. Previous research has suggested that a low rate of omission errors, i.e., failing to press the button in response to the target stimulus, on similar tasks reflect sustained attention, while a high rate of commission errors, i.e., responding to non-target stimuli, reflect disinhibition or impulsivity (Halperin et al., 1988; Barkley, 1991).

***Cognitive flexibility task***. Children were administered a computerized task that required them to use, on a trial-by-trial basis, one of two visually presented rule cues (“Color” or “Direction”) to determine the appropriate response for a given target stimulus (Baym et al., 2008). Target stimuli were cartoons from a popular animated children's film. Color and Direction trials were randomly ordered. On Direction trials, children were instructed to press a left button in response to an arrow that pointed to the left of the screen, and a right button for an arrow that pointed to the right. On Color trials, half of the children were taught that a red stimulus indicated a left-button press and a blue stimulus indicated a right button press, and half were taught the reverse stimulus-response mapping. Trials involved either Incongruent features, in which the color and direction required different responses (e.g., a left-facing stimulus in a color associated with a right-button press), or Congruent features, in which the color and direction required the same response (e.g., a left-facing stimulus in a color associated with a left-button press). Finally, rules for each trial (i.e., Color or Direction) were either the same as on the preceding trial (“Repeat”) or different from the preceding trial (“Switch”).

To ensure that they understood the directions of the task, children participated in a short practice session (32 trials) followed by a testing session (98 trials). On each trial, an instructional cue (“Color” or “Direction”) appeared for 2300 ms, followed by a target stimulus for 1500 ms. Children were required to press the appropriate button while the target stimulus was displayed. If a child responded incorrectly to a stimulus during the practice session, the experimenter would pause the task and remind the child of the correct response. No feedback was given to the child during the testing session. Data was missing for one child on this task due to a computer malfunction. For the present study, proportion correct [100 *(# of correct trials/# of total trials)] on Incongruent-Switch trials (24 total trials) was used as a measure of cognitive flexibility. As measured by this task, cognitive flexibility is a more specific type of interference suppression, as accurate performance on these trials requires suppression of the previously relevant stimulus dimension (i.e., interference suppression), as well as flexible switching from one rule to another (Baym et al., 2008).

#### Parent and teacher questionnaires

The child's participating parent and main classroom teacher completed Attention Focusing and Inhibitory Control subscales of the Children's Behavior Questionnaire (CBQ; Rothbart et al., 2001). Items were rated on a 7-point scale ranging from 1 (*extremely untrue)* to 7 *(extremely true)*. Parent and teacher reports of the two CBQ subscales have been used previously with both European American and Chinese children, and have shown satisfactory internal and test-retest reliabilities in both European American and native Chinese children (Eisenberg et al., 2005; Zhou et al., 2008). Moreover, cross-cultural comparisons between Chinese and US samples showed cross-cultural similarities in the relations of attention focusing and inhibitory control (Zhou et al., 2009). In the present sample, the alphas for parent and teacher-rated inhibitory control (11 items) were 0.74 and 0.81, respectively; the alphas for parent and teacher-rated attention focusing (12 items) were 0.77 and 0.88, respectively. Inhibitory control and attention focusing are two theoretically related components of effortful control (Rothbart and Bates, 2006). Within the present sample, the inhibitory control and attention focusing subscale scores were moderately to highly correlated within reporters, *r*s = 0.65 and 0.71 (*N*s = 216 and 192), for parents' and teachers' reports, respectively, *p*s < 0.001. Thus, following procedures used in previous studies (Olson et al., 2005), an effortful control composite was computed by averaging the item scores across the two subscales.

#### Laboratory tests of language proficiency

***Chinese language proficiency***. Two measures were used to assess children's proficiency in Chinese receptive vocabulary and Chinese character recognition. To assess children's receptive vocabulary, children were administered the Chinese version of the Peabody Picture Vocabulary Test-Revised (Lu and Liu, 1998). To assess children's proficiency in Chinese character recognition, children were presented with a sheet containing 20 Chinese characters of increasing complexity, and asked to identify as many words as they could (Gottardo et al., 2001, 2006). The items are commonly used in texts encountered by beginning readers. Characters were presented in both traditional and simplified Chinese characters. Children were given one point for each correct response, for a maximum of 20 points. Children were given partial credit (0.5 points) if they were unable to provide the Chinese pronunciation for the character, but were able to provide the correct English meaning.

***English proficiency***. The Basic Reading Skills cluster from the Woodcock-Johnson III Tests of Achievement III (WJ III; Woodcock et al., 2001) was used to assess children's English proficiency. The Basic Reading Skills cluster consists of two subtests: Letter-Word Identification (naming and reading words from a list) and Word Attack (the ability to read non-words). The WJ III is standardized with a mean score of 100 and a standard deviation of 15. The age-standardized scores were used in the analyses.

## Results

Analyses were conducted in the following steps. First, all study variables were screened for normality. Second, zero-order correlations were used to examine associations between demographic variables, Chinese and English language proficiencies, and separate measures of self-regulation across laboratory, home, and classroom contexts. Finally, multiple regression analyses were used to examine the unique and interactive associations between children's Chinese and English proficiencies and components of self-regulation.

### Descriptive statistics

Descriptive statistics for all main variables are listed in Table 1. All variables were screened for normality. Based on recommended cutoffs of two and seven for skewness and kurtosis, respectively (West et al., 1995), one variable was positively skewed (most participants made few errors of omission on the response inhibition task), whereas another variable was negatively skewed (most participants showed high levels of behavioral persistence on the puzzle box task). Given the presence of non-normal variables, we conducted the regression analyses using maximum likelihood estimation with robust standard errors (Muthén and Muthén, 1998).

**Table 1:** **Descriptive statistics of main variables**.

	***N***	**Min**	**Max**	**Mean**	***SD***	**Skewness**	**Kurtosis**
Child age (years)	222	7.49	10.96	9.18	0.74	0.08	−0.69
English literacy	223	86	134	108.85	10.43	0.12	−0.43
Chinese literacy	222	0	20	9.88	6.32	−0.05	−1.21
Chinese receptive vocabulary	223	2	119	48.15	22.28	0.26	−0.25
Behavioral persistence	214	0.09	1.00	0.94	0.15	−3.77	15.74
Go/No-Go omission errors	223	0	20	1.82	2.90	2.90	11.00
Go/No-Go commission errors	223	0	34	9.38	5.42	1.16	2.48
Cognitive flexibility	222	0.50	1.00	0.82	0.13	−0.63	−0.29
Parent-rated effortful control	220	2.77	6.59	4.72	0.74	0.22	−0.27
Teacher-rated effortful control	194	2.33	6.96	5.29	0.91	−0.63	−0.01

### Zero-order correlations

Zero-order correlations among main variables are reported in Table 2. Family SES was computed as a composite score by first averaging maternal and paternal education levels and then averaging the standardized scores of parental education and family per capita income. Some associations were found between demographic characteristics and main variables. Older children performed better on some measures of self-regulation (e.g., fewer commission errors, *r* = −0.15, *p* < 0.05, better behavioral regulation, *r* = 0.29, *p <* 0.001) and tests of Chinese proficiency (*r*s between 0.25 and 0.27, *p*s < 0.001). However, older children received lower age-standardized English reading scores (*r* = −0.23, *p* < 0.001). Higher family SES was associated with better English literacy (*r* = 0.22, *p* < 0.01) and higher parent-rated effortful control (*r* = 0.17, *p* < 0.01). Finally, boys exhibited worse performance than girls on most tests of self-regulation (*r*s between −0.13 and −0.41, *p*s < 0.05 to < 0.001). Given these associations, child age, family SES, and child gender were included as covariates in the main analyses.

**Table 2:** **Correlations among main variables**.

	**1**	**2**	**3**	**4**	**5**	**6**	**7**	**8**	**9**	**10**	**11**	**12**
1. Child age	–	−0.15^*^	−0.08	−0.23^***^	0.27^***^	0.25^***^	0.28^***^	−0.17^*^	−0.16^*^	0.15^*^	0.04	0.07
2. Family SES		–	−0.06	0.22^***^	−0.01	−0.02	−0.05	−0.01	0.10	0.09	0.17^*^	0.04
3. Child gender			–	−0.09	−0.11	−0.07	−0.22^***^	−0.05	0.08	−0.13^*^	−0.15^*^	−0.41^***^
4. Literacy (E)				–	0.10	−0.04	0.06	−0.23^**^	0.01	0.09	0.23^***^	0.36^***^
5. Literacy (C)					–	0.52^***^	0.18^**^	−0.002	−0.21^**^	0.11	0.06	0.19^**^
6. Receptive vocabulary (C)						–	0.19^**^	−0.01	−0.10	0.12	0.14^*^	0.23^***^
7. Behavioral persistence							–	−0.05	−0.07	0.12	0.17^*^	0.28^***^
8. Omission errors								–	0.12	−0.17^**^	−0.18^**^	−0.02
9. Commission errors									–	−0.27^***^	−0.09	−0.18^*^
10. Cognitive flexibility										–	0.14^*^	0.23^***^
11. Effortful control (P)											–	0.35^***^
12. Effortful control (T)												–

*** *p ≤ 0.001*,

** *p ≤ 0.01*,

* *p ≤ 0.05, Correlations for non-normally distributed variables (Behavioral persistence and Omission errors) are Spearman correlations. All other correlations are Pearson correlations. SES, socioeconomic status; E, English; C, Chinese; P, parent report; T, teacher report*.

Overall, zero-order correlations also indicated that children's English and Chinese proficiencies were both associated with better self-regulation, though these associations varied across specific measures. Children's English literacy was associated with fewer omission errors on the response inhibition task (*r* = −0.23, *p* < 0.01) and higher parent and teacher ratings of effortful control (*r*s between 0.23 and 0.36, *p*s < 0.01 to < 0.001). On the other hand, Chinese literacy was associated with fewer commission errors on the response inhibition task (*r* = −0.21, *p* < 0.01) and higher teacher-reported effortful control (*r* = 0.19 and 0.15, *p*s < 0.01). Finally, children's receptive Chinese vocabulary was associated with better behavioral regulation on the puzzle task (*r* = 0.21, *p* < 0.01), better cognitive flexibility (*r* = −0.14, *p* < 0.01), and better parent and teacher-rated attention focusing and inhibitory control (*r*s between 0.15 and 0.22, *p*s < 0.10 to < 0.01).

Zero-order correlations also indicated a number of positive associations among measures of self-regulation. With the exception of correlations between parent-reported measures (*r* = 0.65, *p* < 0.001) and between teacher-reported measures (*r* = 0.71, *p* < 0.001), associations among self-regulation measures were in the weak to moderate range (*r*s between 0.15 and 0.33), suggesting that measures represented interrelated but dissociable components of self-regulation.

### Multiple regressions: predicting self-regulation measures from chinese proficiency, english proficiency, and their interactions

Six multiple regression models were specified to predict each of the six self-regulation measures (including four lab measures and two adult-reported measures) from the following predictors: (a) the covariates (child age, child gender, and family SES), (b) Chinese receptive vocabulary, Chinese literacy, and English literacy, and (c) the interactions between Chinese proficiency and English proficiency: Chinese receptive vocabulary × English literacy and Chinese literacy × English literacy. To minimize multicollinearity and aid interpretation (Aiken and West, 1991), the main effect predictors of language proficiency were mean centered prior to computing the interaction terms.

Because of the presence of non-normal variables, the regression models were estimated using the Maximum Likelihood Robust (MLR) estimator in Mplus 6.12 (Muthén and Muthén, 1998). Missing data were handled using the full information maximum likelihood (FIML) estimation approach. The estimates of regression coefficients and *R*^2^ values are reported in Table 3 (for lab-based self-regulation measures) and Table 4 (for adult-reported self-regulation measures). Across models, unique variance contributed by the language predictors ranged from values of *R*^2^ = 0.03 (for language variables predicting behavioral persistence) to *R*^2^ = 0.15 (for language variables predicting teacher-reported effortful control.

**Table 3:** **Multiple regressions predicting lab-based measures of self-regulation from Chinese and English proficiency**.

**Predictors**	**DV: Behavioral persistence**		**DV: Response Inhibition—Commission errors**		**DV: Response Inhibition—Omission errors**		**DV: Cognitive flexibility**	
	**B (*SE*)**	**β**	**B (*SE*)**	**β**	**B (*SE*)**	**β**	**B (*SE*)**	**β**
Child age	5.48^***^ (1.54)	0.27	−0.74 (0.50)	−0.10	−0.74^**^ (0.27)	−0.19	2.23 (1.35)	0.13
Family SES	−1.54 (0.85)	−0.09	0.75 (0.47)	0.11	0.16 (0.22)	0.03	0.96 (0.96)	0.06
Child gender (0 = girls, 1 = boys)	−4.28^*^ (1.76)	−0.15	0.38 (0.71)	0.04	−0.31 (0.36)	−0.05	−1.90 (1.65)	−0.08
Chinese literacy	−0.22 (0.19)	−0.10	−0.16^*^ (0.07)	−0.18	0.04 (0.03)	0.09	0.02 (0.16)	0.01
Chinese receptive vocabulary	0.11^*^ (0.05)	0.17	0.002 (0.017)	0.01	−0.01 (0.01)	−0.09	0.05 (0.04)	0.09
English literacy	0.11 (0.12)	0.08	−0.02 (0.04)	−0.03	−0.08^**^ (0.02)	−0.28	0.15 (0.08)	0.12
Chinese literacy × English literacy	0.01 (0.01)	0.04	−0.01 (0.01)	−0.09	0.01 (0.003)	0.11	0.03^*^ (0.01)	0.16
Chinese receptive vocabulary × English literacy	0.003 (0.01)	0.05	0.001 (0.002)	0.05	0.000 (0.001)	−0.02	−0.01 (0.004)	−0.11
Total *R*^2^		0.14^***^		0.08^*^		0.09^*^		0.08^*^
*R*^2^ from adding language variables		0.03		0.04		0.07^*^		0.04

*** *p ≤ 0.001*,

** *p ≤ 0.01*,

* *p ≤ 0.05*.

**Table 4:** **Multiple regressions predicting adult-reported measures of self-regulation from Chinese and English proficiency**.

**Predictors**	**DV: Parent-reported effortful control**		**DV: Teacher-reported effortful control**	
	**B (*SE*)**	**β**	**B (*SE*)**	**β**
Child age	0.07 (0.07)	0.07	0.12 (0.08)	0.10
Family SES	0.12^*^ (0.06)	0.13	−0.05 (0.07)	−0.04
Child gender (0 = girls, 1 = boys)	−0.18 (0.10)	−0.12	−0.63^***^ (0.11)	−0.35
Chinese literacy	−0.01 (0.01)	−0.07	−0.002 (0.01)	−0.01
Chinese receptive vocabulary	0.01 (0.003)	0.15	0.008^**^ (0.003)	0.21
English literacy	0.01^**^ (0.01)	0.20	0.03^***^ (0.01)	0.36
Chinese literacy × English literacy	0.000 (0.001)	0.01	−0.001 (0.001)	−0.04
Chinese receptive vocabulary × English literacy	0.000 (0.000)	0.02	0.000 (0.000)	−0.03
Total *R*^2^		0.11^**^		0.33^***^
*R*^2^ from adding language variables		0.06^*^		0.15^***^

*** *p ≤ 0.001*,

** *p ≤ 0.01*,

* *p ≤ 0.05*.

Controlling for child age, gender, and family SES, Chinese receptive vocabulary uniquely predicted higher behavioral persistence. Chinese literacy uniquely predicted a lower number of commission errors on the response inhibition task (failures of inhibition to No-Go stimuli), whereas English literacy uniquely predicted a lower number of omission errors on this task (failure to respond to Go stimuli within the allotted time). Finally, English literacy uniquely predicted higher parent-reported effortful control, and both English literacy and Chinese receptive vocabulary uniquely predicted higher teacher-reported effortful control.

**Figure 1:**
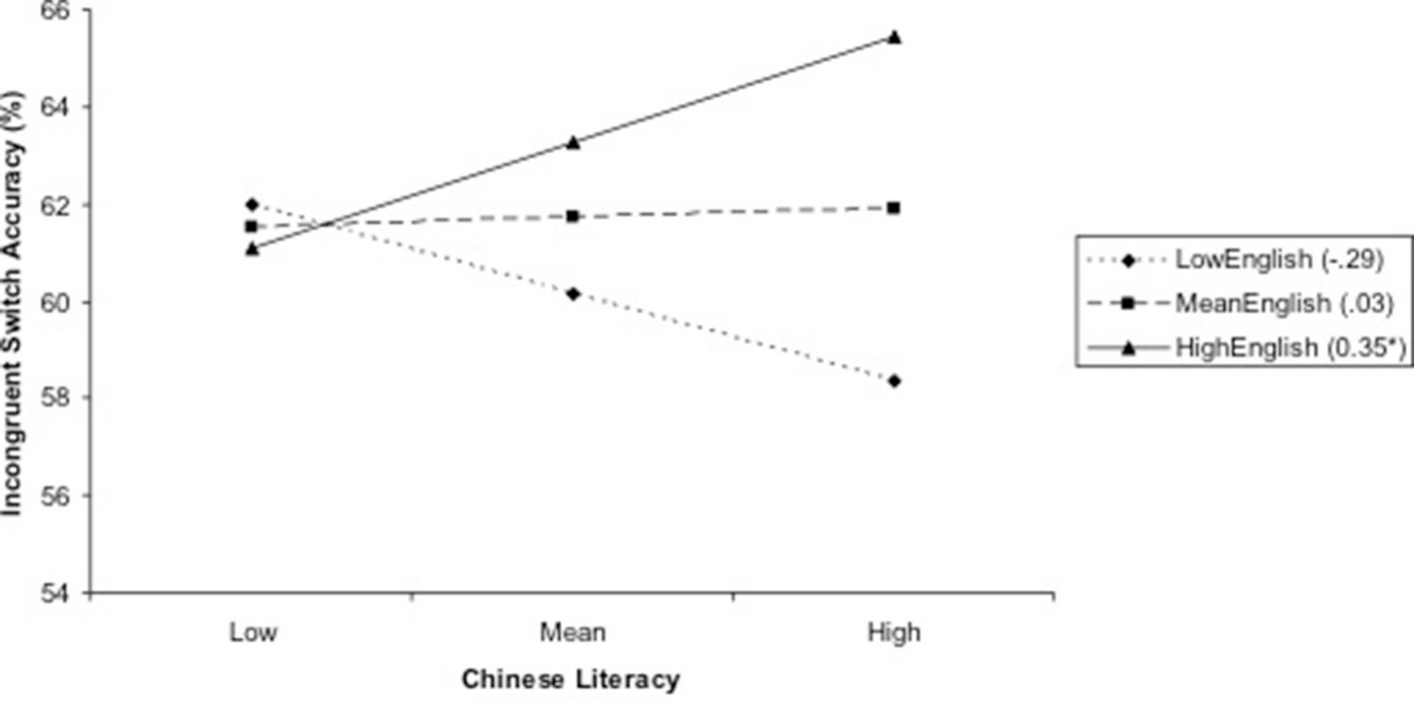
**The interaction of Chinese × English literacy predicting cognitive flexibility**. The numbers in parentheses are unstandardized simple slopes. ^***^p ≤ 0.001; ^**^p ≤ 0.01; ^*^p ≤ 0.05.

For the regression predicting % accuracy on incongruent-switch trials of the cognitive flexibility task, there was a significant interaction effect of Chinese literacy × English literacy. Following the procedures outlined by Aiken and West (1991), simple slope analyses were conducted to probe the significant interaction effect (i.e., Chinese literacy × English literacy predicting cognitive flexibility). In simple slope analysis, the relations between Chinese literacy and cognitive flexibility (% accuracy on the incongruent-switch trials of the cognitive flexibility task) were estimated at three levels of English literacy: mean level, one standard deviation above the mean (“high”), and one standard deviation below the mean (“low”), controlling for other predictors in the model. At low and mean levels of English literacy, Chinese literacy was non-significantly associated with the cognitive flexibility score (i.e., unstandardized simple slopes were non-significant); at high levels of English literacy, Chinese literacy was positively related to cognitive flexibility, the unstandardized simple slope (*SE*) = 0.35 (6.0), *p* = 0.02 (Figure 1). Thus, cognitive flexibility scores were positively associated with children's bilingual literacy, but only for children with higher levels of proficiency in both English and Chinese.

## Discussion

The present study examined potential variations on the bilingual advantage in executive function in an ethnically homogeneous sample of bilingual children, and in doing so extended previous research in three critical ways. First, our study provides only limited evidence that the bilingual advantage in executive function may be observed on a continuum among children with varying levels of exposure and proficiency in two languages. Second, by utilizing a multi-method, multi-informant assessment of self-regulation as a broad construct, our study suggests that the bilingual advantage in executive function may be limited primarily to lab-based cognitive tasks of executive function, while higher proficiency in Chinese or English may be uniquely associated with children's self-regulatory skills in home and school contexts. Third, by recruiting a diverse socioeconomic sample of Chinese American immigrant families, we were also able to identify the unique contributions of both socioeconomic and linguistic factors to children's self-regulation. Within our sample, family SES was positively associated with children's English literacy as well as parent-rated effortful control. However, even when family SES was included as a covariate, our analyses still indicated significant contributions of child language proficiencies across different domains of self-regulation.

### Key findings and future directions

Results from the present study both expand and refine previous findings on bilingualism and executive function. Namely, our results provide limited evidence that bilingualism-related advantages in executive function may be observed on a continuum among dual language learners, but only on laboratory-based tests of cognitive flexibility, and only among children with highest levels of bilingual proficiency. Consistent with previous findings (Martin-Rhee and Bialystok, 2008), children high in both English and Chinese proficiencies did not perform better on the Go/No-Go task, which involved inhibition of a response to a single-feature, or univalent display.

Unexpectedly, higher bilingual proficiency was not significantly associated with advantages in other domains of self-regulation. Rather, children's higher proficiency in either Chinese or English was associated with better effortful control, better performance on a task of behavioral persistence, and fewer errors on the Go/No-Go task. As such, our findings suggest that previous cross-cultural differences in executive function observed between Asian and European American children (Sabbagh et al., 2006; Oh and Lewis, 2008; Lan et al., 2011) may also be observed as variations within Asian American immigrant children.

Our findings also highlight directions for future investigations that examine constructs not specified in our original hypotheses. For example, further consideration of children's reaction time on the Go vs. No-Go trials may also yield more nuanced interpretations of our findings. *Post-hoc* analyses indicated that children's faster overall reaction time on the Go/No-Go task was associated with higher English proficiency (*r* = −0.14, *p* = 0.032) and more commission errors (*r* = −0.37, *p* = 0.000). In contrast, slower overall reaction times were associated with more omission errors (*r* = 0.43, *p* = 0.000). Taken together, these preliminary results suggest that higher acculturation among immigrant children (as reflected by higher English proficiency) may also be associated with faster, and possibly more erroneous response tendencies.

These results should be considered in light of the small effect sizes observed across models. Previous meta-analyses on the cognitive benefits of bilingualism indicate a range of small to large effect sizes across studies, and suggest that these effect sizes may be larger in studies using quasi-experimental designs (i.e., comparisons of bilinguals and monolinguals) (Adesope et al., 2010). Indeed, results of the present study are comparable to effect sizes found in other non-experimental studies examining predictors of children's self-regulatory processes (e.g., Lengua et al., 2007; Bernier et al., 2010). Moreover, as language proficiency may be only one of a number of family-level processes that may contribute to self-regulatory capacities in immigrant children (Chen et al., 2014), findings from the present study need to be replicated among other bilingual immigrant samples, and across different developmental periods.

Future research can also help to identify the unique contributions of bilingualism and general verbal ability to different components of children's self-regulation. For example, it is possible that general verbal ability, rather than bilingualism, may facilitate children's use of certain self-regulatory strategies and behaviors, such as support seeking or self-distraction (Roben et al., 2013). Alternatively, as measures of verbal ability have been positively associated with some measures of executive function (Hughes, 1998; Ardila et al., 2000), it is also possible that bilingual advantages on these tasks may be mediated by verbal ability. More specifically, future research is also needed to identify how aspects of language proficiency—i.e., receptive vocabulary, literacy, and productive vocabulary—contribute uniquely to components of self-regulation. Indeed, a limitation of the present study is that measures of Chinese and English proficiency were not directly equivalent (e.g., receptive vocabulary was assessed only in Chinese).

The cross-sectional design of the present study also limits interpretation of the directionality of relations between language and self-regulation. For example, children's higher fluency in Chinese language may reflect greater adherence to traditional Chinese values of behavioral control and self-restraint. These values, in turn, may be reflected in the associations between Chinese proficiency and lower commission errors on the Go/No-Go Task. Moreover, while the aim of this study was to examine potential effects of children's language proficiencies on self-regulation, there is also evidence supporting reciprocal relations—i.e., that children's self-regulatory mechanisms may foster the development of language (Blair and Razza, 2007; see Eisenberg et al., 2005, for a review). This influence may be particularly relevant for children of immigrant parents, and may also explain the positive associations between children's English proficiency, lower omission errors and effortful control, which were not predicted by our hypotheses. The ability to focus attention and persist in the face of distraction are likely to be key skills in children's acquisition of a new language, whether learning language of a new host country, or maintaining their family's heritage language. As such, future longitudinal research is needed to examine the reciprocal, prospective relations between children's dual language proficiencies and their self-regulation.

In sum, findings from the present study underscore both the unique and interactive contributions of English and Chinese proficiency to Chinese American children's self-regulatory capacities. More broadly, our results build on previous research highlighting associations between bilingualism and Asian American immigrant children's positive developmental outcomes (Han, 2010), and suggest that children's self-regulatory capacities may be a key mediator of these associations.

### Conflict of interest statement

The authors declare that the research was conducted in the absence of any commercial or financial relationships that could be construed as a potential conflict of interest.
